# Targeting the PI3K/Akt/mTOR pathway in non‐small cell lung cancer (NSCLC)

**DOI:** 10.1111/1759-7714.13328

**Published:** 2020-01-27

**Authors:** Aaron C. Tan

**Affiliations:** ^1^ Division of Medical Oncology National Cancer Centre Singapore Singapore

**Keywords:** Akt pathway, mTOR pathway, non‐small cell lung cancer, PI3K pathway, targeted therapy

## Abstract

The traditional classification of lung cancer into small cell lung cancer and non‐small cell lung cancer (NSCLC) has been transformed with the increased understanding of the molecular alterations and genomic biomarkers that drive the development of lung cancer. Increased activation of the phosphatidylinositol 3‐kinase (PI3K)/Akt/mechanistic target of rapamycin (mTOR) pathway leads to numerous hallmarks of cancer and this pathway represents an attractive target for novel anticancer therapies. In NSCLC, the PI3K/Akt/mTOR pathway has been heavily implicated in both tumorigenesis and the progression of disease. A number of specific inhibitors of PI3K, Akt and mTOR are currently under development and in various stages of preclinical investigation and in early phase clinical trials for NSCLC. Early evidence has yielded disappointing results. Clinical trials, however, have been performed on predominantly molecularly unselected populations, and patient enrichment strategies using high‐precision predictive biomarkers in future trials will increase the likelihood of success. A greater understanding of the underlying molecular biology including epigenetic alterations is also crucial to allow for the detection of appropriate biomarkers and guide combination approaches.

## Introduction

Lung cancer remains one of the leading causes of cancer death worldwide.^1^ The traditional classification of lung cancer into small cell lung cancer and non‐small cell lung cancer (NSCLC) has been transformed with the increased understanding of the molecular alterations and genomic biomarkers that drive the development of lung cancer. Targeted therapies for epidermal growth factor receptor (*EGFR*), anaplastic lymphoma kinase (*ALK*), *ROS1* and *BRAF* have resulted in marked improvements in survival, particularly for patients with advanced disease.^2^ Increased activation of the phosphatidylinositol 3‐kinase (PI3K)/Akt/mechanistic target of rapamycin (mTOR) pathway leads to numerous hallmarks of cancer, including acquired growth signal autonomy, inhibition of apoptosis, sustained angiogenesis, increased tissue invasion and metastasis and insensitivity to antigrowth signals. Consequently, this pathway represents an attractive target for novel anticancer therapies.

## Basic biology of the PI3K/Akt/mTOR pathway

The PI3K/Akt/mTOR pathway and signaling cascade is crucial in the regulation of cellular growth and metabolism. The importance of PI3K in cancer was initially described in 1985 after it was implicated in association with polyoma middle‐T antigen, which is required for tumorigenesis in animals.^3^ Subsequent work has intimately characterized the PI3K signaling pathway, and demonstrated that upregulation of this complex pathway is central in the development of cancer.

PI3Ks are a family of intracellular lipid kinases which phosphorylate the 3′‐hydroxyl group of phosphatidylinositol and phosphoinositides.^4^ They are divided into three classes (I–III), which each have distinct roles in signal transduction. Class I PI3Ks are divided into class IA PI3Ks that are activated by growth factor receptor tyrosine kinases, and class IB PI3Ks that are activated by G‐protein‐coupled receptors.^5^ Class IA PI3K is a heterodimer consisting of a p85 regulatory subunit and a p110 catalytic subunit. The p85 regulatory subunit is encoded by the *PIK3R1*, *PIK3R2* and *PIK3R3* genes which encode the p85α, p85β and p55γ isoforms, respectively, and the p110 catalytic subunit is encoded by the *PIK3CA*, *PIK3CB* and *PIK3CD* genes which encode the p110α, p110β and p110δ isoforms, respectively.^6^ Class II PI3Ks consist of a p110‐like catalytic subunit only. The *PIK3C2A*, *PIK3C2B* and *PIK3C2G* genes encode the PIK3C2α, PIK3C2β, PIK3C2γ isoforms, respectively. Class III PI3K consists of a single catalytic member, vacuolar protein sorting 34 (Vps34), which is encoded by the *PIK3C3* gene. Vps34 binds to the adapter protein Vps15, which is encoded by the *PIK3R4* gene.^7^

The role of each class of PI3K can be generally categorized into their importance in cell signaling (class I and II) or membrane trafficking (class II and III). A majority of the evidence for the importance of PI3K in human cancer implicates class IA PI3Ks, and specifically the p110α isoform. The presence of *PIK3CA* gene mutations or amplifications has been found in a diverse range of malignancies.^8^ In a breast cancer mouse model, inhibition of the p110α isoform led to increased mammary tumorigenesis.^9^ Preclinical evidence has also identified a modulatory or regulatory role for other class IA isoforms such as p110β and p110δ.^9^, ^10^ Further preclinical data suggests that there exists significant functional redundancy of class IA PI3Ks, and only a small fraction of total class I PI3K activity is required to maintain cell survival and proliferation.^11^ Inhibition of specific PI3K isoforms, such as p110α, may also lead to the upregulation of alternative bypass pathways such as the ERK pathway. Class IA PI3Ks can be activated by upstream receptor tyrosine kinases and growth factor stimulation. The regulatory subunit of the PI3K binds to the receptor tyrosine kinase and leads to the release of the p110 catalytic subunit, which translocates to the plasma membrane.^12^ PI3K phosphorylates phosphatidylinositol 4,5‐bisphosphate (PIP2), to produce PI(3,4,5)P_3_ (PIP3).^13^ Phosphate and tensin homolog (PTEN) can regulate this step by dephosphorylating PIP3 to PIP2 and preventing further signal transduction.^14^ Activated PIP3 allows for Akt activation via phosphorylation by phosphoinositide‐dependent kinase‐1 (PDK1), and consequently loss of PTEN is a key mechanism by which cancers increase PI3K signaling.^15^ Germline mutations of *PTEN* as seen in Cowden syndrome also result in high risk of numerous cancers including breast, thyroid, endometrial and genitourinary cancers.^16^

Akt is a member of the AGC (PKA/PKG/PKC) protein kinase family and consists of three homologues, Akt1, Akt2 and Akt3 located at chromosomes 14q32, 19q13 and 1q44, respectively.^17^ Akt activation subsequently leads to a number of potential downstream effects. It can result in inhibition of BAD and BAX, proapoptotic Bcl2 family members. Akt may also phosphorylate Mdm2, which causes downregulation of p53‐mediated apoptosis and forkhead transcription factors that produce cell‐death promoting proteins.^5^ The nuclear factor kappa‐light‐chain‐enhancer of activated B cells (NFκB) transcription factor plays a crucial role in the consequences of PI3K/Akt pathway activation. NFκB regulates gene expression of hundreds of genes which are implicated in apoptosis, cell cycle control, immune modulation, cell survival and cell adhesion and differentiation.^18^ Akt prevents negative regulation of NFκB by the IκB family, and in particular IκBα. IκBα removes NFκB from DNA and returns it to the cytoplasm.^19^ Another important downstream pathway resulting from Akt activation is activation of the protein kinase, mTOR. This occurs via the phosphorylation of TSC2, which subsequently activates Rheb which then stimulates the multiprotein complex mTORC1. mTORC1 leads to further downstream activation of the eIF4 complex and subsequent promotion of tumorigenesis, regulation of the cell cycle and inhibition of apoptosis.^4^ Another mTOR complex, mTORC2, causes Akt activation through phosphorylation with serine 473.^20^

The importance of upregulation, increased activation and constitutive signaling of the PI3K/Akt/mTOR pathway in cancer is well documented. Somatic mutations that encode for the various components of the signaling cascade and gene amplifications have been demonstrated in numerous different cancers. Mutations in *PIK3CA* have been identified in up to 36% of hepatocellular cancers, 26% of breast and 26% of colon cancers.^8^, ^21^ Smaller rates of *PIK3CA* mutations are also seen in ovarian, gliomas, gastric and lung cancers.^8^, ^21^, ^22^, ^23^ These mutations are predominantly clustered to two regions in exon 9 and 20, with exon 20 encoding the catalytic domain of p110α and exon 9 encoding the helical domain of p110α.^24^ *PIK3CA* amplifications are also common, particularly in squamous cell carcinomas, with amplifications in up to 69% of cervical cancers, 66% of SCC of the lung and 42% of head and neck cancers.^25^, ^26^ These amplifications have also been demonstrated in significant proportions of gastric, thyroid, breast, esophageal adenocarcinoma and lung adenocarcinomas.^27^, ^28^, ^29^, ^30^ PTEN loss of heterozygosity is extremely common in glioblastoma (54%), prostate (35%), breast (23%), melanoma (37%) and gastric (47%) cancers, with *PTEN* mutations also seen in the same cancers to a lesser extent.^27^, ^31^, ^32^, ^33^ *Akt* amplifications meanwhile have been identified in head and neck, gastric, pancreatic, ovarian and breast cancers.^34^, ^35^ Gene mutations in the Akt family which encode Akt1, Akt2 and Akt3 have also been demonstrated, and may lead to constitutive membrane localization of Akt.^36^

## Importance of the PI3K/Akt/mTOR pathway in NSCLC

In NSCLC, the PI3K/Akt/mTOR pathway has been heavily implicated in both tumorigenesis and the progression of disease. Somatic mutations of *PIK3CA* and amplifications of *PIK3CA* are frequently found in patients with NSCLC. A large study of 1144 consecutive NSCLC patients investigated tumor tissue using next generation sequencing (NGS) for *PIK3CA* mutations.^37^ Mutations were identified in 3.7% of patients, with predominance for squamous cell carcinoma (8.9%) compared with adenocarcinoma (2.9%). E545K exon 9 mutations (57.1%) were most common, followed by H1047R exon 20 (16.7%) and E542K exon 9 (14.3%) mutations. Importantly, in a significant proportion of patients (57.1%), this study found coexisting oncogenic mutations in genes encoding for *EGFR*, *BRAF*, *ALK* and *KRAS*. Other studies have shown similar findings. Yamamoto *et al*.^38^ examined 86 NSCLC cell lines and 356 resected NSCLC tumors for *PIK3CA* mutations in exon 9 or 20 and *PIK3CA* amplification. Either a mutation or amplification was detected in 12.8% of cell lines and 19.1% of tumors. In this study, *PIK3CA* amplifications were also more common in patients with squamous cell carcinoma (33.1%) compared with adenocarcinoma (6.2%). The functional importance of *PIK3CA* mutation or amplification was confirmed with increased Akt activity, and mutations were similarly not mutually exclusive with *EGFR* or *KRAS* mutations. Further evidence supports the greater presence of *PI3KCA* amplification compared with mutation, and predominantly in patients of squamous cell carcinoma histology.^39^, ^40^, ^41^

Homozygous and heterozygous deletions of *PTEN* have also been shown in lung cancer. Loss of PTEN expression assessed by immunohistochemistry has been demonstrated in up to 24% of 125 resected early stage NSCLC specimens.^42^ This may be in part due to increases in promoter methylation. Another series of early stage NSCLC specimens revealed complete loss of PTEN expression in 44% of tumors, reduced level of expression in 29% and normal expression in 27%.^43^ This study similarly showed methylation of PTEN in 26% of tumors with loss of heterozygosity at microsatellites in chromosome 10q23 occurring in 19% of studied specimens; however, neither was a significant predictor of PTEN protein expression. A retrospective analysis of the phase III FLEX study of chemotherapy in combination with cetuximab in patients with EGFR‐expressing advanced NSCLC, showed 35% with negative PTEN expression.^44^ The presence of PTEN expression is potentially correlated with improved survival. Other studies have also shown total loss of PTEN expression may be more common in squamous cell carcinomas compared with adenocarcinomas.^45^

Upregulation of the Akt pathway has also been demonstrated in a significant proportion of patients with NSCLC. A study of 110 NSCLC tumors revealed 51% with increased Akt activity determined by immunohistochemistry.^46^ There was also significant association with Akt activation and increased mTOR and forkhead activity, important downstream targets of Akt. Malanga *et al*.^47^ examined 105 resected NSCLC specimens, and identified a somatic mutation of the gene encoding for *Akt1* through direct sequencing of PCR products. Two squamous cell carcinoma specimens were found to contain the E17K mutation of *Akt1* in exon 4. Another study using a high resolution melting assay, revealed four of 219 NSCLC specimens with an *Akt1* mutation.^48^ Of these, one contained the E17K mutations and was of squamous cell carcinoma histology. The other three tumors showed rare single nucleotide polymorphisms. The relatively rarity of the E17K mutation in NSCLC has been further confirmed in other series.^49^ The discordance between levels of Akt overexpression and presence of somatic mutation may indicate coexisting mutations or amplifications that result in Akt activation. This has been demonstrated in preclinical studies in NSCLC cell lines, with Akt activation attributed to loss of *PTEN*, *EGFR* or *PIK3CA* mutation, or *HER2* amplification.^50^

Upregulation of the mTOR pathway has also been illustrated in significant proportions of NSCLC tumors, with increased p‐mTOR in up to 90% of patients with adenocarcinoma, 60% of patients with large cell carcinoma and 40% of patients with squamous cell carcinoma.^51^, ^52^, ^53^ Downstream products of mTOR activation, S6K and 4E‐BP1 have also been identified in up to 58% and 25% of NSCLC specimens respectively, with a greater predominance in adenocarcinoma.^54^ There is also a strong correlation of p‐S6K and p‐mTOR positivity. The importance of mTOR in bronchial adenocarcinoma may be related to its coupling with eIF‐4E which functions as an oncogene.^55^ The presence of mTOR activity may also be a poor prognostic factor in early stage NSCLC. Several studies have demonstrated increased mTOR expression as determined by immunohistochemistry was associated with poor survival.^56^, ^57^

The importance of the PI3K/Akt/mTOR pathway also extends to its role in tumors with other known activating mutations, such as *EGFR* and *KRAS*. Studies suggest that in patients with an *EGFR* mutation, the Akt/mTOR pathway is constitutively activated in 67% of cases.^58^ Additionally, another study revealed 18% of specimens showed positive staining for p‐EGFR, p‐Akt and p‐mTOR, indicating the importance of this signaling cascade.^52^ The importance of Akt activation may also relate to the development of resistance to therapy. Preclinical evidence suggests that Akt activation may confer acquired resistance to EGFR inhibitors in *EGFR* mutant NSCLC.^59^ Similarly mTOR activation has been associated with *EGFR* and *KRAS* mutations and may act as a mechanism of resistance to EGFR inhibitors.^60^ Given the encompassing downstream signaling effects of the PI3K/Akt/mTOR pathway on the development and progression of malignancy, and its potential influence in response and resistance to standard therapies, it represents an attractive target for anticancer therapy in NSCLC.

## Clinical trials of PI3K/Akt/mTOR inhibitors in NSCLC

A number of specific inhibitors of PI3K, Akt and mTOR are currently under development and in various stages of preclinical investigation and in early phase clinical trials for NSCLC (Table 1). There are several pan‐class I PI3K inhibitors, including pictilisib (GDC‐0941), PX‐866, buparlisib (BKM120), pilaralisib (XL‐147) and GNE‐317. Pictilisib has been evaluated in phase I trials either alone or in combination with standard chemotherapy. In a phase IA dose‐escalation trial of patients with advanced solid tumors, the single‐agent maximum tolerated dose was 330 mg, with maculopapular rash as the dose limiting toxicity.^83^ Pictilisib has been further investigated in a phase IB dose‐escalation trial in combination with standard first‐line chemotherapy in patients with advanced NSCLC.^61^ Patients received pictilisib with either carboplatin and paclitaxel or cisplatin and pemetrexed, with the addition of bevacizumab depending on histology. Of 66 patients, 29 (43.9%) had partial response and 20 (30.9%) had stable disease. This led to the phase II FIGARO study of pictilisib in combination with first‐line chemotherapy; however, preliminary data did not reveal any significant progression‐free survival (PFS) or overall survival (OS) benefit.^84^ Similar findings have been reported in a phase IA/IB trial in a Japanese cohort of patients.^85^ PX‐866 has been evaluated in combination with docetaxel chemotherapy in a randomized phase II trial of advanced, refractory NSCLC patients.^62^ There was no improvement in PFS, response rate or OS with the addition of PX‐866. These patients, however, were molecularly unselected. In the phase I trial of PX‐866 in combination with docetaxel, there was one NSCLC patient with a *PIK3CA* mutation who had a prolonged response to continuation PX‐866 after cessation of docetaxel.^86^ Buparlisib has also been investigated as monotherapy in the BASALT‐1 phase II trial of previously treated patients with NSCLC.^63^ Patients were selected based on identification of PI3K pathway activation. The primary objective for efficacy based on 12‐week PFS, was not met. Further studies of buparlisib in combination with chemotherapy were similarly negative,^64^ whilst it has also been studied in combination with gefitinib.^65^ Pilaralisib has also been assessed as monotherapy in phase I trials, with one partial response in a patient with NSCLC.^66^ Another phase I trial in combination with erlotinib established its safety profile although similarly exhibited only one partial response.^61^

**Table 1:** PI3K/Akt/mTOR inhibitors evaluated in clinical trials for NSCLC

Drug	Phase of development for NSCLC	References
**Pan‐PI3K inhibitors**		
Pictilisib (GDC‐0941)	II	Soria *et al*. 61
PX‐866	II	Levy *et al*. 62
Buparlisib (BKM120)	II	Vansteenkiste *et al*.^63^ Adjei et al.^64^ Tan *et al*.^65^
Pilaralisib (XL‐147)	I	Soria *et al*.^61^ Shapiro *et al*.^66^
**Selective PI3K inhibitors**		
Alpelisib (BYL719)	II	Zhou *et al*.^67^
INK1117	I	Iartchouk *et al*. 68
GSK2636771	I	Mateo *et al*.^69^
AZD8186	I	Siu *et al*.^70^
SAR260301	I	Bedard *et al*. 71
Taselisib (GDC‐0032)	II	Langer *et al*.^72^
**Akt inhibitors**		
Perifosine	II	Lara *et al*.^73^
**mTOR inhibitors**		
Everolimus	II	Besse *et al*. 74 Price *et al*.^75^
Sirolimus	II	Moran *et al*.^76^ Blumenthal *et al*.^77^
Temsirolimus	II	Gandhi *et al*.^78^
Ridaforolimus	II	Riely *et al*.^79^
**Dual PI3K/mTOR inhibitors**		
BEZ235	II	Herrera *et al*.^80^
XL765	I	Cohen *et al*.^81^
GDC‐0980	I	Calvo *et al*.^82^

Whilst the pan‐class I PI3K inhibitors have shown disappointing efficacy, there are isoform‐specific class I PI3K inhibitors also in development. Alpelisib (BYL719) is a potent p110α inhibitor, and is currently under investigation in a phase II study of patients with advanced NSCLC and a *PIK3CA* mutation or amplification.^67^ INK1117 is another p110α‐specific inhibitor in early phase development.^68^ Specific p110β inhibitors such as GSK2636771,^69^ AZD8186^70^ and SAR260301^71^ have also been tested in phase I trials in patients with advanced solid tumors including NSCLC. Taselisib (GDC‐0032) is a PI3K inhibitor that inhibits the p110α, p110γ and p110δ isoforms, but spares the p110β isoform. It has been evaluated as part of the phase II LUNG‐MAP study, in previously treated NSCLC patients with a *PIK3CA* mutation.^72^ This substudy was closed for futility after an interim analysis.

Perifosine is an Akt inhibitor that has been investigated in a phase I trial of patients with advanced NSCLC.^87^ Of 15 patients evaluated, there was one unconfirmed partial response. The trial was expanded to a phase II trial, the results of which have not yet been reported. MK‐2206 is a highly selective Akt inhibitor which was evaluated in a phase II trial, in combination with erlotinib, in NSCLC patients who had previously progressed on erlotinib therapy.^73^ Patients were stratified based on *EGFR* mutation status. Median PFS was 4.4 months in *EGFR* mutant patients, and 4.6 months in *EGFR* wild‐type patients.

mTOR inhibitors are widely used to prevent transplant rejection, and are approved as anticancer therapy for renal cell carcinoma, neuroendocrine tumors and breast cancer. Everolimus is an mTOR inhibitor that selectively inhibits mTORC1 signaling. It has been assessed in several phase I trials in previously treated advanced NSCLC, either as monotherapy^88^ or in combination with pemetrexed chemotherapy.^89^ The combination of everolimus and EGFR inhibitors has also been evaluated in phase II trials in NSCLC patients unselected for *EGFR* mutation status. Everolimus in combination with erlotinib^74^ or gefitinib,^75^ both failed to show significant efficacy to progress to a phase III trial. Sirolimus, another oral mTOR inhibitor, has demonstrated significant toxicity in combination with afatinib;^76^ however, showed potential activity in combination with pemetrexed chemotherapy.^77^ Trials of mTOR inhibitors in molecularly selected patients have also been conducted. Temsirolimus, an intravenous mTOR inhibitor has also been evaluated in a phase II trial in combination with neratinib, an oral HER2 inhibitor, in *HER2* mutant advanced NSCLC patients.^78^ The combination therapy resulted in a 19% response rate. Ridaforolimus has also been investigated in *KRAS* mutant advanced NSCLC patients in a phase II trial versus placebo, with a two month improvement in PFS.^79^

Second generation inhibitors have also been developed with dual targeting of both PI3K and mTOR. Importantly, these agents block both mTORC1 and mTORC2 complexes, and have pan‐class I PI3K inhibitory activity. BEZ235 is one such agent, currently under evaluation in numerous phase I/II clinical trials, and preclinical evidence suggests strong activity in lung cancer cell lines.^80^ XL765 has been tested in a phase I trial in combination with erlotinib with a cohort of patients with NSCLC, and the combination was generally well tolerated.^81^ GDC‐0980 has also been investigated in combination with chemotherapy in a phase Ib trial with a NSCLC cohort with the trial illustrating an acceptable safety profile.^82^ Vistusertib (AZD2014) in combination with paclitaxel demonstrated a 33% response rate in previously treated squamous NSCLC, in the dose escalation arm of the TAX‐TORC study.^90^

Finally, there are also dual PI3K/mTOR inhibitors that have been developed. Gedatolisib has been evaluated in a phase I trial in combination with chemotherapy (docetaxel or cisplatin) in NSCLC patients or dacomitinib in *EGFR* mutant NSCLC patients.^91^ Toxicity profiles were manageable, and ongoing phase I/II trials are underway.

## Conclusion

Early evidence for targeted therapies against the PI3K/Akt/mTOR pathway have yielded disappointing results. Clinical trials, however, have been performed on predominantly molecularly unselected populations, and patient enrichment strategies using high‐precision predictive biomarkers in future trials will increase the likelihood of success. A greater understanding of the underlying molecular biology, including epigenetic alterations is also crucial to allow for the detection of appropriate biomarkers and guide combination approaches. Furthermore, innovative and novel clinical trial design will enhance our ability to evaluate novel agents and combinations to account for molecular diversity and ultimately improve patient outcomes.

## Disclosure

The author declares no conflict of interest. A.C.T is the recipient of an International Association for the Study of Lung Cancer (IASLC) Fellowship 2018‐2020.
